# Supplementary material for: Evidence supported by Mendelian randomization: impact on inflammatory factors in knee osteoarthritis
Source: Front Med (Lausanne). 2024 May 28;11:1382836. doi: 10.3389/fmed.2024.1382836 (PMC11165061; doi:10.3389/fmed.2024.1382836)
Supplement: Supplementary file 1 [file Data_Sheet_1.ZIP › data/GCST90274815_5e6/815.forest.pdf]

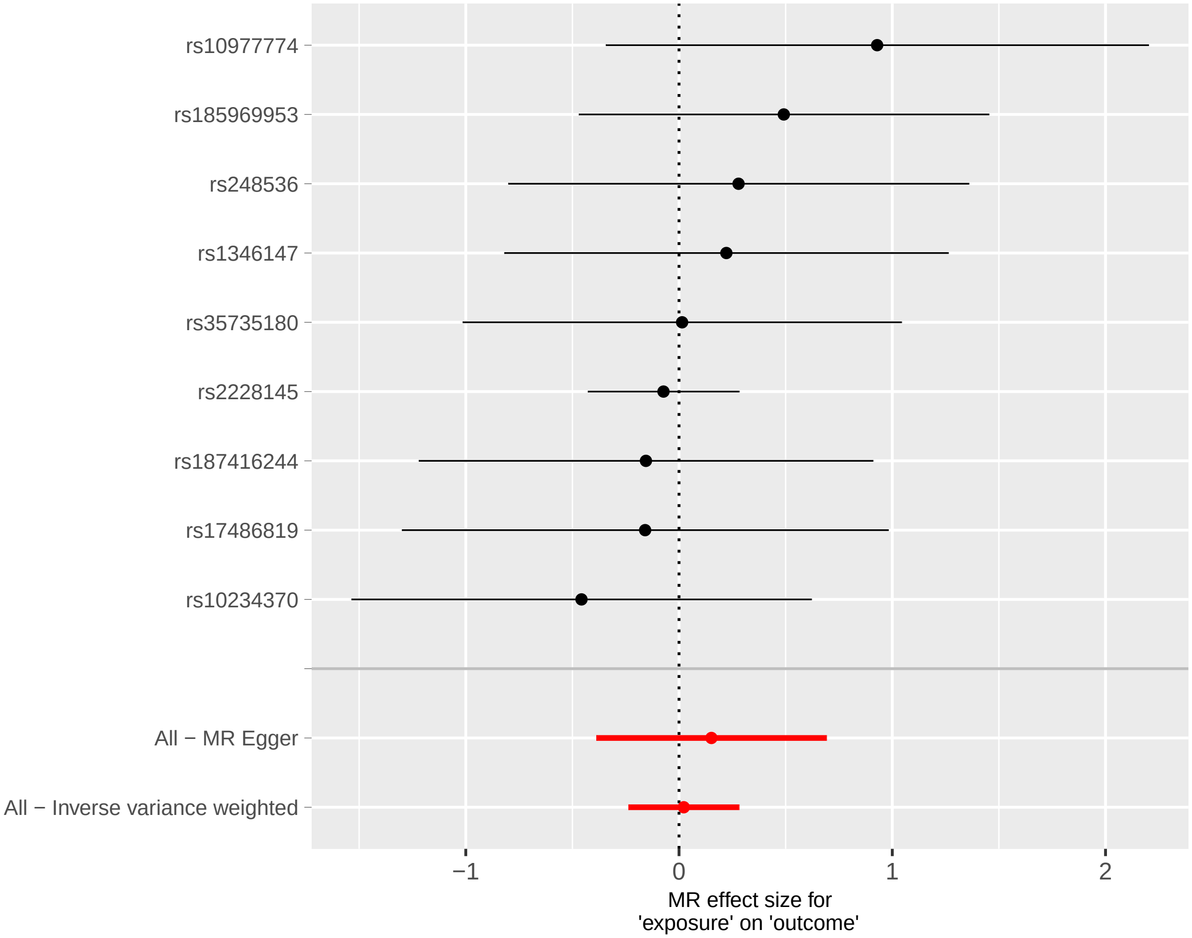
rs10977774
rs185969953
rs248536
rs1346147
rs35735180
rs2228145
rs187416244
rs17486819
rs10234370
All − MR Egger
All − Inverse variance weighted
−1
0
1
2
MR effect size for
'exposure' on 'outcome'
